# Physical performance, health-related quality of life and sleepiness severity of an adult outpatient population with narcolepsy: A cross-sectional analysis

**DOI:** 10.1016/j.ijchp.2025.100573

**Published:** 2025-05-02

**Authors:** R. Tadrous, D. O’ Rourke, N. Murphy, G. Quinn, L. Slattery, J. Broderick

**Affiliations:** a Discipline of Physiotherapy, School of Medicine, Trinity College, Dublin, Ireland; b Department of Neurology, St. James’s Hospital, Dublin, Ireland; c Department of Physiotherapy, St. James’s Hospital, Dublin, Ireland

**Keywords:** Narcolepsy, Hypersomnolence, Physical activity, Exercise, Quality of life, Sedentary behaviour

## Abstract

**Objectives:**

This study aimed to profile and explore the correlation between health-related quality of life (HRQoL), sleepiness severity and physical performance in a sample of people with narcolepsy type 1 (NT1).

**Methods:**

This cross-sectional study took place in a dedicated outpatient narcolepsy clinic. The following variables were evaluated: cardiopulmonary fitness, physical activity, muscle strength, muscle endurance, power. HRQoL was assessed using the Short Form-36 (SF-36) and the Functional Outcome of Sleep Questionnaire (FOSQ). Sleepiness Severity was measured by the Epworth Sleepiness Scale (ESS), and symptom severity was assessed using the Narcolepsy Severity Scale (NSS).

**Results:**

A total of 22 participants (31.53 ± 13.17 years, 56.52 % female) completed the test battery. Physical performance was low across all measures. With the exception of accelerometery (54.17 % compliance), there was full compliance (100 %) with the test battery, indicating its feasibility for people with narcolepsy. Results showed that physical performance and HRQoL were markedly reduced compared to normative values in this sample of people with narcolepsy. Self-reported physical activity was associated with higher health-related quality of life (ρ = 0.41; *p* = 0.05), and greater moderate-vigorous physical activity was associated with higher physical wellbeing (ρ = 0.58; *p* = 0.04). Higher BMI was associated with significantly lower predicted VO₂ Max (0.001), and wall sit duration (*p* = 0.039).

**Conclusion:**

The influence of physical performance on sleepiness severity and quality of life warrants further investigation, including the possible physical rehabilitation strategies to target physical performance deficits.

## Introduction

Narcolepsy is a chronic sleep disorder characterised by excessive daytime sleepiness and is frequently associated with episodic muscular weakness, known as cataplexy. Narcolepsy exerts a significant burden on physical wellbeing, with physical role limitations and vitality identified as the most affected quality of life domains (Tadrous et al., 2021). With significant correlations identified between health-related quality of life (HRQoL) and sleepiness severity (Dauvilliers et al., 2017), a closer evaluation of how physical function (such as cardiorespiratory fitness, strength and endurance) is impacted in people with Narcolepsy (pwN) may be warranted.

Physical function is described as the “ability to execute daily activities with optimal fitness, endurance, and strength with the management of disease, fatigue, stress and reduced sedentary behaviour”(Campbell et al., 2013). It is a multidimensional construct including the components of cardiorespiratory fitness, muscular strength, muscular endurance, flexibility and body composition(Britton et al., 2020), and is routinely measured objectively through physical performance tests(van Lummel et al., 2015). The relationship between physical functioning and sleepiness severity in people with narcolepsy is not fully understood and is likely complex. One study identified that poorer cardiorespiratory fitness in pwN correlated with increased severity of excessive daytime sleepiness and the frequency of cataplexy attacks (Matoulek et al., 2017). In children with narcolepsy, a positive correlation was identified between increased physical activity and lower body mass index (BMI), increased night-time sleep duration, higher sleep quality, and reduced frequency of napping (Filardi et al., 2018). Considerably lower physical activity has been reported in pwN than in the general population (Parmar et al., 2019), and this reduced physical activity has been shown to correlate with depressive symptoms (Bruck et al., 2005). Additionally, bidirectional relationships have been identified between exercise and sleep, with exercise acting as an effective nonpharmacological treatment option for disturbed sleep, and poor sleep contributing to lower physical activity levels (Kline, 2014). The potential use of exercise to modulate sleepiness severity and quality of life in pwN remains underexplored.

However, little is known about the physical performance of pwN. In other populations with chronic conditions, profiling physical performance variables such as strength, cardiopulmonary fitness, and physical activity can provide early indications of increased risk of functional decline, hospitalisation and mortality (Corsonello et al., 2012; Legrand et al., 2014). Profiling the physical performance of pwN may help identify individuals that are at risk of functional decline at an earlier time point, as their performance on physical assessments fell below established normative thresholds or demonstrated significant deviations from age- and sex-matched population data. Identifying the physical performance of pwN could help tailor exercise recommendations for this cohort and aid the exploration of how physical performance influences the quality of life and symptom severity of pwN. The objectives of this study were to profile the physical performance and HRQoL of pwN and explore the correlation between physical performance, HRQoL and sleepiness severity.

### Methods

This cross-sectional mixed-method study took place in a dedicated narcolepsy outpatient clinic at St James's Hospital Study method and results are reported following the Strengthening the Reporting of Observational Studies in Epidemiology (STROBE) Statement for cross-sectional studies (Von Elm et al., 2007). Ethical approval for this study was obtained from the St. James’s Hospital/Tallaght University Hospital Research Ethics Committee (REC: 2019-09 List 35 (06); clinicaltrials.gov registration: NCT04419792).

### Study procedure

Potentially eligible participants were screened in advance of their routine clinic visits and a participant information leaflet was sent. Participants were required to meet the following eligibility criteria: aged 18 to 65 years, diagnosed with type 1 narcolepsy based on the International Classification of Sleep Disorders third edition criteria (American Academy of Sleep Medicine, 2014) for at least six months, eligibility screened by their treating clinician, and able to understand English and follow simple instructions to enable completion of assessments. Individuals with sleep disorders other than narcolepsy, uncontrolled or severe comorbid medical conditions (e.g., cardiovascular disease, uncontrolled diabetes) that could interfere with participation, contraindications to moderate-intensity exercise, confirmed pregnancy, or significant psychiatric illness or cognitive impairment were excluded from participating in the study.

A post hoc power analysis was conducted using SPSS (Version 29) to evaluate the statistical power of a one-sample *t*-test based on the actual sample size. An a priori analysis indicated that a sample size of 27 participants would be required to achieve a power of 0.812, assuming a medium effect size (Cohen’s *d* = 0.5) and a significance level of α = 0.05. However, due to recruitment limitations, the final sample consisted of 22 participants, resulting in an achieved power of 0.734. It is likely that with a power of 0.734, there is a reasonable probability of correctly rejecting the null hypothesis that there is no difference between the study sample and population averages.

During their clinic visit, the study assessor (RT) provided additional study-related information and answered any questions. Following the obtainment of consent, the following demographic information was collected: age, gender, narcolepsy subtype, living arrangements, employment status and highest educational achievement. Participants then underwent the battery of physical performance outcome measures described below in a single assessment session. Participants received an ActiGraph accelerometer and were instructed on its use. Participants were provided with an envelope to return the accelerometer following one week of continuous wear. The assessment process lasted approximately 45 min and the following outcome measures were completed:

1. To assess cardiopulmonary endurance, the YMCA submaximal bike test was utilised to estimate VO_2_ max. The test is designed to raise the steady-state heart rate to between 110 beats per minute and 85 % of their age-predicted maximal heart rate for at least two consecutive stages (American College of Sports Medicine, 2013). The YMCA submaximal bike test is reported to have a moderately high correlation coefficient of *r* = 0.79, and when used to assess cardiopulmonary fitness in a heterogeneous population, Beekley et al. (2004) found no statistical difference between the predicted VO_2_ max and the criterion measure (mean difference = 1.3 ml/kg^-1^/min).
2. To objectively measure physical activity and sedentary behaviour levels, ActiGraph accelerometry was employed. Participants were asked to wear an ActiGraph for seven consecutive days, excluding swimming or bathing. ActiGraph data was downloaded and analysed using ActiLife Software (ActiGraph Manufacturing Technology Inc., FL). Subjective physical activity was measured by the Physical Activity Vital Sign questionnaire (Greenwood et al., 2010).
3. To estimate upper limb strength, grip strength was assessed using dynamometry. Measurements were obtained in standardised conditions (JAMAR, Hatfield, PA, USA). The Jamar dynamometer has excellent test-retest reliability (ICC = 0.822), and interrater reliability (ICC = 0.996–0.998) as reported by Mathiowetz et al. (1984), and (Lindstrom-Hazel et al., 2009), respectively.
4. To ascertain upper limb endurance, the American College of Sports Medicine (ACSM) Press up test was employed. The number of push-ups performed consecutively without rest was counted. The test was stopped when the participant strained visibly or was unable to maintain the appropriate technique within two repetitions (American College of Sports Medicine, 2013). The Push-Up test has a test-retest interclass correlation coefficient of 0.95, with a 95 % confidence interval of 0.85–0.99 (Ryman Augustsson et al., 2009).
5. To measure lower limb endurance, the Isometric Wall Sit test was utilised. Participants were timed from the moment they obtained the proper test position until they could no longer maintain this position (Tomchuk, 2011). The intra-class correlation coefficient for the wall squat test ranges from 0.69 to 0.88 (Lubans et al., 2011).
6. To evaluate the vertical jump height and power of the lower limbs, the Countermovement Jump test was used. The best of three trials was recorded to the nearest 0.5 inches or 1.0 cm (Haff & Triplett, 2015). When compared to other jump tests, the countermovement jump test is the most reliable measure of lower-body power (Markovic et al., 2004). Furthermore, the countermovement jump test demonstrates great factorial validity through its relationship with explosive power (*r* = 0.87), low within-subject variation of 2.8 % and high reliability with a Cronbach’s alpha of 0.98 (Markovic et al., 2004).

### Threshold classifications

Threshold classifications for each physical performance measure were based on normative data and stratified according to age and sex. Predicted VO_2_ max thresholds followed recommendations from the American College of Sports Medicine (2013), whereas handgrip strength thresholds were provided by Dodds et al. (2014). Push-up performance was interpreted using the American College of Sports Medicine (2013) guidelines, and countermovement jump thresholds were obtained from Markovic et al. (2004). For the isometric wall sit test, normative values were obtained from McIntosh et al., 1998).

### Health-Related quality of life and symptom severity

HRQoL was evaluated using the Medical Outcomes Short-Form 36 (SF-36) and the functional outcomes of daytime sleepiness questionnaire (FOSQ).

The SF36 is the most widely used scale for measuring HRQoL in pwN (Tadrous et al., 2021), and it has been used in various populations and different health conditions (Ware, 2000). The SF-36 includes one multi-item scale that assesses eight health domains: Physical Functioning, Physical Role Limitations, Bodily Pain, General Health, Vitality, Social Function, Emotional Role Limitations and Mental Health. A higher score implies better health status. These eight domains can be combined into a physical component score (PCS) and a mental component score (MCS) to provide a general overview of health and wellbeing (Ware & Sherbourne, 1992). The SF-36 is a validated and reliable instrument for assessing HRQoL, with strong internal consistency (Cronbach’s α > 0.80 for most subscales) and good test-retest reliability (ICCs ranging from 0.60 to 0.90) (McHorney et al., 1993; Ware & Sherbourne, 1992). The SF-36, however, may lack the specificity to assess the subtle aspects of the HRQoL imposed by narcolepsy (Tadrous et al., 2021).

The FOSQ takes approximately 15 min to complete and measures how a person’s daily ability to function is affected by their sleepiness. This is conceptually defined as everyday behaviours encompassing the areas of physical, mental, and social functioning in daily life (Weaver et al., 1997). The FOSQ contains five domains: General Productivity, Social Outcome, Activity Levels, Vigilance and Sexuality/Intimacy. Domain scores can be summated, and a total score can be calculated. The FOSQ has an internal reliability of *a* = 0.95 and a test–retest reliability ranging from 0.81 to 0.90 (Weaver et al., 1997). The Epworth Sleepiness Scale (ESS) is an eight-item questionnaire that measures the general level of daytime sleepiness in adults. Respondents are asked to distinguish dozing behaviour from tiredness and rate the likelihood of falling asleep in specific situations using a 4-point Likert scale. Higher scores indicate greater sleepiness, with scores above 10 suggesting excessive daytime sleepiness (Johns, 1991)**.** The ESS has good internal consistency (Cronbach’s α = 0.73–0.88) and strong test-retest reliability (ICCs = 0.82–0.87) in both clinical and general populations (Johns, 1991, 1992).

The Narcolepsy Severity Scale (NSS) is a 15-item scale that assesses the clinical symptoms of narcolepsy such as EDS, cataplexy, hallucinations, sleep paralysis and disturbed night-time sleep. The NSS has high internal consistency, strong test-retest reliability, and good construct validity, making it useful for monitoring symptom progression and treatment response (Dauvilliers et al., 2017).

### Statistical analysis

Data was entered into Excel, checked and coded. The physical performance variables of participants within this study were descriptively quantified. Normality was assessed using the Kolmogorov-Smirnov test. As all data was non-normally distributed, only non-parametric statistics were applied. The correlation between HRQoL (as measured by the SF-36 PCS and MCS scores, and FOSQ total score), physical performance variables (subjective physical activity (mins/day), predicted VO2 Max (mL.kg.min-1), MVPA (mins/day) and objective sedentary behaviour (hours/day)) and sleepiness severity (as measured by the ESS) were explored using Spearman correlation coefficients. A p-value of <0.05 was considered statistically significant. The data obtained from the study was analysed using IBM SPSS V26 software. Missing data were not imputed due to the percentage of missing data. Instead, a complete case analysis was undertaken as analyses were conducted using only the data available from participants who provided valid actigraphy recordings. As such, outcome measures related to actigraphy reflect this subset of the total sample.

## Results

**Fig. 1:**
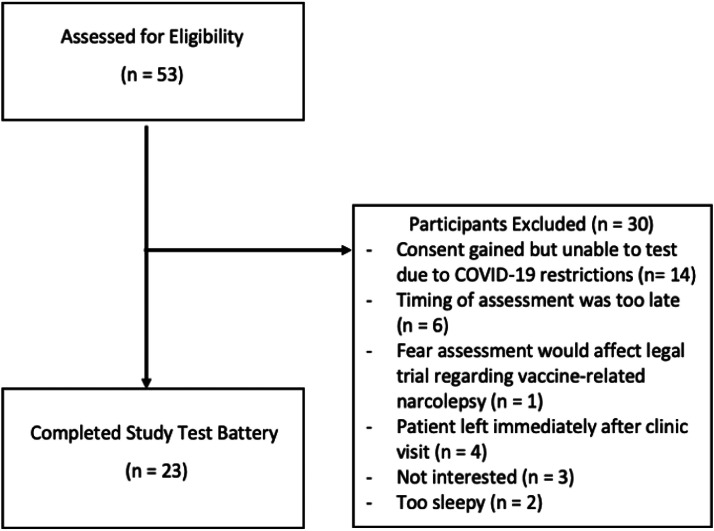
Flow diagram of participants through the study.

A total of 22 pwN with type 1 narcolepsy participated (Fig. 1). Recruitment and testing for this study ceased early on March 7th, 2020 due to Covid-19 restrictions. The demographic characteristics of participants are provided in Table 1. Just over half of the sample were female (*n* = 12, 56.52 %). The mean age of participants was 31.4 (±13.2) years, with an age range of 20–63 years. The majority of participants lived with their families (*n* = 19, 86.36 %).

**Table 1:** Demographic characteristics of participants.

Demographics	N	%
**Gender**		
Males	10	45.45
Females	12	54.55
**Age**		
Age, Years Mean (SD)	31.4 ± 13.2	
Age, Years Range	20 - 63	
Median Age	25	
**Living Arrangements**		
Alone	1	4.55
Partner	2	9.01
Family	19	86.36
**Highest Educational Achievement**		
Second Level	12	54.55
Third Level	10	45.45
**Current Education/Employment status**		
Currently Enrolled as a Student	10	45.45
Employed	9	40.91
Unemployed/Retired	3	12.64
**Medications (*n*=37)**		
Modafinil	12	32.43
Venlafaxine	9	24.32
Wakix	5	13.51
Concerta	4	10.81
Ritalin	4	10.81
Other	3	8.12

### Feasibility of test battery

Actigraphy was obtained for 13 participants, resulting in an adherence rate of 54.17 % for this measure. The non-compliance was due to a mix of equipment shortages and COVID-19 restrictions preventing follow-up. There was a completion rate of 100 % for all other components of the test battery.

### Physical performance

The results of the test battery are presented below in Table 2, with threshold classifications provided in Supplemental Table 1.

1. **Aerobic Capacity**: The collective group median (IQR) predicted VO 2 max for this population was
38.8 (10.8) mL.kg.min −1 .
2. **Actigraphy**: The median (IQR) daily duration spent sedentary by participants over the measurement period was 9.1 (11.7) hours, with prolonged bouts of sedentary behaviour ranging from 10 min to 22.8 hours. The mean length of a sedentary bout was 35.95 (± 18.83) minutes. The levels of sedentary behaviour observed in female participants (15.0 (8.6) hours were higher than that observed in male participants (3.6 (7.1) hours). The time spent in moderate-vigorous physical activity per day was 42.20 ± 21.41 min daily and the mean daily step count was 3949.84 ± 2133.75 steps.
3. **Dynamometry**: The median (IQR) grip strength of the sample was 33.6 (7.5) kg.
4. **ACSM Press Up Test**: The median (IQR) number of push-ups performed by the participants was 10.0 (10.0) repetitions, and the number of push-ups completed ranged from 1 to 32.
5. **Countermovement Jump Test:** The median (IQR) peak power/body mass (W/Kg) of the sample
was 41.8 (10.6) W/kg.
6. **Wall Squat Test**: The median (IQR) duration of the participant’s performance was 44.9 (36.3) seconds, with a range of 9.84 to 122.00 s.

**Table 2:** Summary of physical performance measures.

Outcome Measure	All Participants	N	Male Participants	N	Female Participants	N	P Value
Predicted VO_2_ Max, mL.kg.min-1, median (IQR)	39.0 (10.8)	22	39.3 (9.5)	10	37.4 (16.9)	12	0.69
Moderate-Vigorous Physical Activity, mins/day, median (IQR)	37.3 (31.2)	13	46.8 (32.9)	6	37.3 (17.3)	6	0.52
Objective Sedentary Behaviour, hours/day, median (IQR)	9.1 (11.7)	13	3.6 (7.1)	6	15.0 (8.6)	6	0.04
Hand Dynamometry, kg, median (IQR)	33.6 (17.5)	22	44.2 (7.1)	10	23.9 (3.7)	12	<0.001
Press Up Repetitions, n, median (IQR)	10.0 (10.0)	22	10.0 (9.3)	10	8.0 (9.0)	12	0.13
Peak Power/Body Mass, W/Kg, median (IQR)	41.8 (10.6)	22	43.2 (20.3)	10	41.4 (6.6)	12	0.20
Wall Sit Duration, seconds, median (IQR)	44.9 (36.3)	22	52.0 (30.2)	10	36.3 (39.2)	12	0.43

### Health-Related quality of life and sleepiness severity

The pooled mean results of the SF-36 domains are reported with 95 % confidence intervals in Table 3. From the obtained results, the mental component summary scores (38.13 ± 11.20) were lower than the physical component summary scores (46.32 ± 8.44). The most affected domains of the SF-36 were Vitality (37.04 ± 22.53), Physical Role Limitations (55.30 ± 25.50) and perceived General Health (55.30 ± 25.50). The least affected domains were Physical Functioning (76.74 ± 17.81), and Pain (73.39 ± 26.56).

**Table 3:** Pooled mean results for short form 36.

	PF	RP	BP	GH	PCS	V	SF	RE	MH	MCS
**Pooled Mean**	76.74	44.57	73.39	55.30	46.32	37.04	59.80	53.94	66.00	38.13
Standard Deviation	17.81	38.40	26.56	25.50	8.44	22.53	27.19	42.46	17.59	11.20
**Males (mean)**	82.00	50.00	85.00	56.00	48.14	47.00	65.00	60.00	71.60	41.45
Standard Deviation	17.83	39.09	17.72	25.91	7.39	17.19	21.08	46.62	15.83	10.67
**Females (mean)**	72.69	40.38	64.46	54.77	44.92	29.38	55.81	49.28	61.69	35.58
Standard Deviation	17.39	38.92	29.31	26.23	9.22	23.72	31.34	40.27	18.25	11.33

PF; Physical Function, RP; Physical Role Limitations, BP; Bodily Pain, GH; General Health, PCS; Physical Component Summary.

V; Vitality, SF; Social Functioning, RE; Emotional Role Limitations, MH; Mental Health, MCS: Mental Component Summary.

The scores for the FOSQ domains and standard deviations are reported in Table 4. From the results obtained, Vigilance (48.50 ± 15.67) and Activity Levels (54.72 ± 15.49) were identified as the most affected domains of HRQoL, whereas Intimacy/Sexuality (58.26 ± 23.48) and Social Outcomes (59.57 ± 21.21) were the least affected FOSQ domains.

**Table 4:** Mean results for functional outcome of sleep questionnaire.

	General Productivity	Social Outcome	Activity Levels	Vigilance	Intimacy/Sexual	Total Score
**Pooled Mean**	57.37	59.57	54.72	48.50	58.26	68.83
Standard Deviation	14.29	21.21	15.49	15.67	23.48	17.80
**Males (mean)**	60.75	63.00	58.33	50.07	62.00	73.54
Standard Deviation	14.46	18.89	13.10	16.46	22.01	19.95
**Females (mean)**	54.76	56.92	51.94	47.29	55.38	65.21
Standard Deviation	14.17	23.23	17.09	15.60	25.04	15.80

**Fig. 2:**
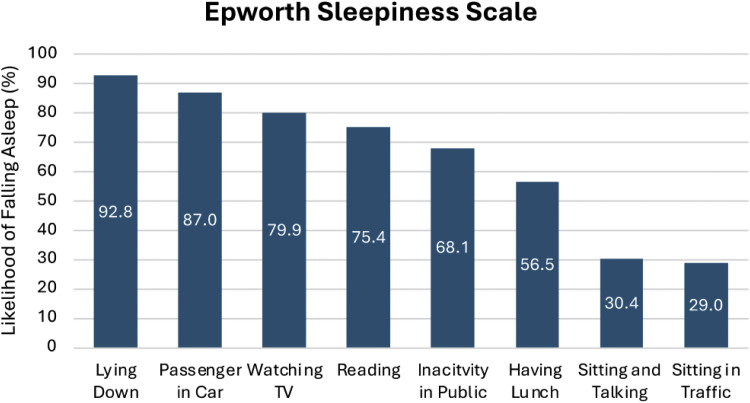
Activities likely to cause sleep as measured by the epworth sleepiness scale.

All participants completed the ESS. Scores were summated, and the total score obtained was found to be 64.86 ± 19.09. The activities which were most likely to lead to sleeping were lying down (92.75 ± 17.28) and being a passenger in a car (86.96 ± 24.08). The activities least likely to lead to sleeping were sitting and talking (30.43 ± 31.64) and sitting in traffic whilst driving (28.99 ± 19.00). The scores for each activity of the ESS are outlined below in Fig. 2.

**Fig. 3:**
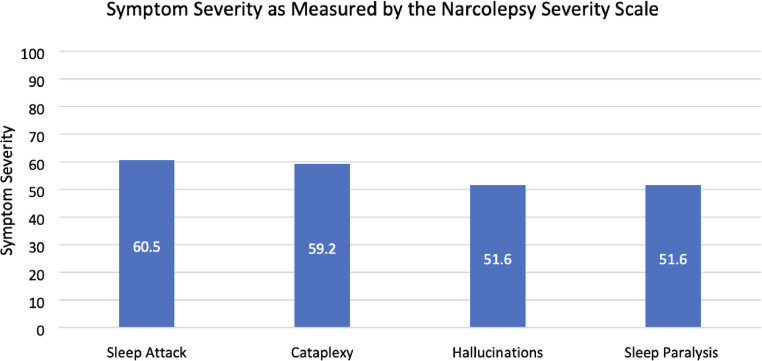
Bar chart of symptom severity as measured by the narcolepsy severity scale.

**Fig. 4:**
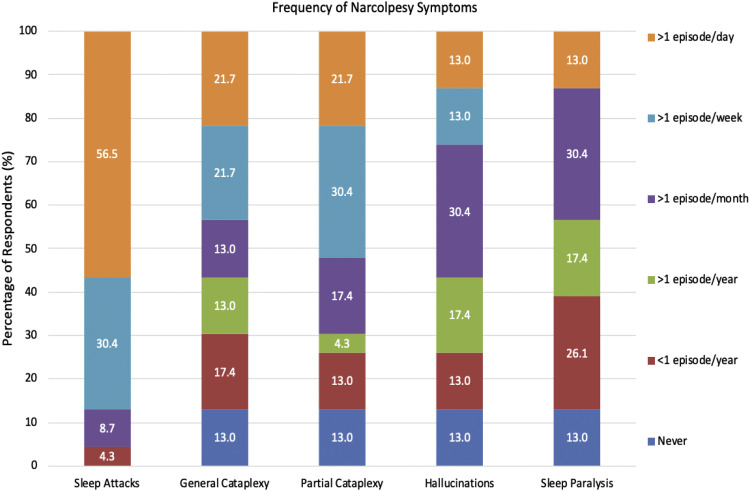
Bar chart of the frequency of narcolepsy symptoms.

All 22 participants completed the Narcolepsy Severity scale. Sleep attacks were identified as the most severe symptom of narcolepsy (60.5 ± 22.4), followed closely by cataplexy (59.2 ± 30.7). Sleep paralysis (51.6 ± 22.6), and hallucinations (51.6 ± 25.4) were found to be the least severe symptoms (Fig. 3). Sleep attacks were the most frequently experienced symptom, occurring daily for 59 % of respondents (*n* = 13). General and Partial Cataplexy were both experienced daily in 22.7 % of participants (*n* = 5). Sleep paralysis and hallucinations were the least frequently occurring symptoms, both occurring in only 13 % of respondents daily, respectively. Symptom frequency is reported in Fig. 4.

## Correlation analysis

The relationship between physical performance, HRQoL and sleepiness severity was explored through Spearman’s rank-order correlation analyses. The results show that greater subjective physical activity as measured by the Physical Activity Vital Sign was associated with higher HRQoL as measured by the FOSQ (ρ = 0.41; *p* = 0.05). Similarly, greater daily MVPA was associated with higher physical wellbeing as measured by the physical component summary of the SF-36 (ρ = 0.58; *p* = 0.04). The remaining associations are provided below in Table 5. A multi-variate regression analysis was also conducted to explore the relationship between BMI and physical performance, HRQoL and sleepiness severity (Table 6). Higher BMI was significantly associated with lower predicted VO₂ Max (β (−0.632) and significant p-value (0.001), and shorter wall sit duration (*p* = 0.039). The relationship between BMI and the remaining outcome measures were not statistically significant.

**Table 5:** Correlation between physical performance measures and health-related quality of life and epworth sleepiness scale scores.

	N	SF-36 PCS		SF-36 MCS		FOSQ Total Score		ESS Total Score	
		Spearman ρ	P	Spearman ρ	P	Spearman ρ	P	Spearman ρ	P
Subjective Physical Activity (mins/day)	23	0.01	0.95	−0.03	0.89	0.41	0.05*	0.13	0.57
Predicted VO2 Max, mL.kg.min-1	23	0.06	0.8	0.07	0.75	−0.13	0.55	0.05	0.81
Moderate-Vigorous Physical Activity (mins/day)	13	0.58	0.04*	−0.17	0.58	0.52	0.07	−0.45	0.12
Objective Sedentary Behaviour, hours/day	13	0.11	0.72	−0.38	0.19	0.02	0.94	−0.22	0.47

SF-36; Short Form-36; PCS; Physical Component Summary, FOSQ; Functional Outcome of Sleep Questionnaire, ESS; Epworth Sleepiness Scale.

**Table 6:** Multiple regression analysis of BMI and physical performance, symptom severity, and health-related quality of life.

Outcome Variable	B	SE	β	t	p	R²	Adj. R²
Subjective Physical Activity (mins/day)	−2.843	166.816	−0.118	−0.545	0.591	0.118	−0.033
VO₂ Max (mL.kg.min⁻¹)	−1.000	8.55733	−0.632	−3.740	**0.001**	0.632	0.371
Hand Dynamometry, kg, median (IQR)	−0.135	10.03158	−0.093	−0.430	0.672	0.093	−0.038
Press Up Repetitions, n, median (IQR)	−0.456	8.75450	−0.342	−1.665	0.111	0.342	0.075
Peak Power/Body Mass, W/Kg, median (IQR)	0.040	8.72061	0.032	0.146	0.885	0.032	−0.047
Wall Sit Duration, seconds, median (IQR)	−3.007	43.69585	−0.433	−2.202	**0.039**	0.433	0.149
SF-36 PCS (Physical Component Score)	−0.349	8.29292	−0.282	−1.346	0.193	0.282	0.036
SF-36 MCS (Mental Component Score)	−0.409	11.10472	−0.249	−1.180	0.251	0.249	0.017
NSS Total Score	0.176	10.21224	0.119	0.550	0.588	0.119	−0.033
FOSQ Total Score	0.038	18.21623	0.015	0.067	0.947	0.015	−0.047
ESS Total Score (Sleepiness Severity)	−0.100	4.637	−0.148	−0.688	0.499	0.148	−0.025

## Discussion

To our knowledge, this is the first study to use a comprehensive test battery to assess physical performance in an adult outpatient population with narcolepsy. Our results indicate poor physical performance in the sample. Furthermore, the HRQoL observed in this sample was comparable to the pooled values for pwN observed in an earlier meta-analysis as measured by the SF-36 (Tadrous et al., 2021). Female participants largely had lower physical performance than men. A possible explanation for this decline in wellbeing could be the prolonged diagnostic delay in women compared to men, with 85 % of men likely to receive a diagnosis within 16 years after symptom onset, compared to 28 years in women, despite similar symptom presentation (Won et al., 2014). Additionally, women experience more severe sleepiness and symptom-burden than men, which echoes similarities observed in the general population, or may be attributable to suboptimal management of NT1, or worse depressive symptoms in women (Ingravallo et al., 2024).

This study contributes to the literature exploring the interrelationship between physical performance and HRQoL in pwN. Fatigue, reduced vitality, and physical role limitations are central to the lived experience in pwN and are the most affected domains of HRQoL (Tadrous et al., 2021). Fatigue and reduced vitality have been associated with functional impairment, depressive symptoms and lower quality of life in this population (Droogleever Fortuyn et al., 2012). Physical performance in pwN has been suggested to affect mental wellbeing (Morse & Sanjeev, 2018), with lower physical activity levels in this population being linked to poorer mood (Bruck et al., 2005). PwN often have reduced opportunities to engage in physical activity owing to commonly cited barriers such as sleepiness and social isolation (Kapella et al., 2015), and oftentimes have to prioritise catching up on work (Tadrous et al., 2023). This reduced physical activity is associated with increased sleepiness severity(Golden & Lipford, 2018), which in turn may increase symptom burden, making it more challenging to engage in physical activity and exercise (Tadrous et al., 2023) and further reduce habitual physical activity levels and worsen HRQoL (Matoulek et al., 2017). The impact of physical activity on physical and mental wellbeing in this population warrants further exploration.

Participants had higher predicted mean VO_2_ max (39.0 mL.kg.min-1 ± 34.3) than the cohort assessed in the study by Matoulek et al. (2017) (30.1 mL.kg.min-1 ± 7.5). However, the present study estimated cardiopulmonary fitness using a less robust submaximal test, while the latter used maximal cardiopulmonary exercise testing so these results should be cautiously interpreted. Notably, the predicted VO_2_ max of participants in our study was generally below average when compared to matched normative values. As cardiopulmonary fitness is inversely correlated to sleepiness severity and cataplexy episodes per month (Matoulek et al., 2017), and improves levels of plasma orexin-A(Messina et al., 2016), further research is warranted to explore the relationship between symptoms and cardiopulmonary fitness in pwN.

Similar levels of moderate-vigorous physical activity were observed in our sample (42.20 ± 21.41 min daily) as the unmedicated (42.51 ± 10.33 min) and medicated (49.44 ± 13.57 min) pwN in a previous study by Bruck et al. (2005). Additionally, we found the duration of MVPA in this study to be above the recommended threshold of 150–300 min of moderate-intensity physical activity per week (Bull et al., 2020). Considerably fewer steps were observed in this sample (3949.84 ± 2133.75 steps) than those obtained by adolescents with narcolepsy (7808.7 ± 3089.5), with Parmar et al. identifying that lower self-reported physical activity was associated with higher depressive symptoms (Parmar et al., 2019). In this study, higher subjective physical activity was associated higher HRQoL as measured by the FOSQ (ρ = 0.41; *p* = 0.05)., whereas higher MVPA was associated with improved physical wellbeing as measured by the SF-36 (ρ = 0.58; *p* = 0.04). This reduced physical activity and high levels of sedentary behaviour (9.1 ± 11.7 h) can have deleterious impacts on pwN and have been linked with higher body mass index, reduced night-sleep quality and duration, and increased frequency of napping (Filardi et al., 2018). The sedentary behaviour time for female participants (>15 hours per day) appeared particularly high. Prolonged sedentary behaviour has been shown to correlate with poorer mental wellbeing in pwN (Parmar et al., 2019), and hypertension (Beunza et al., 2007), high blood glucose (Dempsey et al., 2014), physical inactivity (Van Der Ploeg et al., 2010) and obesity (Shields & Tremblay, 2008) in the general population. Strategies to address prolonged sedentary behaviour and physical inactivity to improve HRQoL in this population may be warranted.

Participants had reduced lower limb strength and endurance as measured by the wall squat test and CMJ test, with the peak power/body mass lower than matched normative values(Tsubaki et al., 2009). The grip strength of our study sample was also lower than matched normative values (Steiber, 2016). This deserves consideration as grip strength correlates with all-cause mortality and mortality from cardiovascular disease, respiratory diseases and different cancers(Celis-Morales et al., 2018). Similarly, push up capacity was markedly reduced when compared to matched normative values(American College of Sports Medicine, 2013). Push up capacity is inversely related to the risk of cardiovascular disease, with individuals able to perform 11 or more push-ups having a significantly lower risk of future cardiovascular events(Yang et al., 2019), yet two-thirds of participants failed to meet this threshold. Further research is necessary to explore the impact of this reduced muscular strength and endurance in pwN.

This study provided a unique insight into the physical performance of pwN using a broad and inclusive test battery. This study proved the feasibility of the test battery, although low compliance with Actigraphy (54.17 %, *n* = 13) was observed. As this was due to a combination of unexpected equipment shortages and COVID-19 precautions, it is likely that incorporating this measure into future studies may work well. This study was limited by a small sample size, as data collection and recruitment ceased twelve weeks early due to COVID-19 restrictions imposed in March 2020, which limits the generalisability of findings. An additional 14 participants had consented but were unable to be assessed due to the aforementioned precautions and the inability to apply the test battery remotely. Furthermore, the decision to opt for submaximal exercise testing limited this study, as direct VO_2_ max testing is the most accurate method of assessing cardiopulmonary fitness. However, the YMCA submaximal exercise test may be more accessible to perform in routine practice, as VO_2_ max testing is more expensive, time-consuming, requires a well-equipped laboratory, highly trained assessors and medical supervision for particular populations (Beekley et al., 2004). Given the nature of the study, selection bias must be considered, as participants may be more physically active. Additionally, self-report bias must also be considered with certain outcome measures such as the SF-36 and FOSQ.

In conclusion, this study found markedly low levels of physical performance and HRQoL in pwN, and further evaluation through larger studies is warranted. The test battery was acceptable and can be easily replicated in future studies. The exploration and development of possible rehabilitation strategies to improve physical performance in this population is also warranted. Given that narcolepsy is particularly challenging to manage from a rest-activity point of view, clinicians should carefully advise pwN to try to break up sedentary behaviour, build sustainable physical activity and structured exercise into their daily routine for long-term health benefits. Further research is required to evaluate the modulating effects of physical activity on sleepiness severity and HRQoL for pwN.

## Clinical trial information

**Name: 'A Profile of Physical Performance Variables in an Out-patient Adult Population With Narcolepsy'.** Registration: NCT04419792 **URL:** https://clinicaltrials.gov/study/NCT04419792

## CRediT authorship contribution statement

**R. Tadrous:** Investigation, Methodology, Project administration, Writing – original draft, Writing – review & editing. **D. O’ Rourke:** Conceptualization, Methodology. **N. Murphy:** Conceptualization, Funding acquisition, Resources. **G. Quinn:** Writing – review & editing. **L. Slattery:** Project administration. **J. Broderick:** Conceptualization, Methodology, Writing – review & editing.

## Declaration of competing interest

The authors declare the following financial interests/personal relationships which may be considered as potential competing interests: Ragy Tadrous reports financial support and equipment, drugs, or supplies were provided by St James’s Hospital. If there are other authors, they declare that they have no known competing financial interests or personal relationships that could have appeared to influence the work reported in this paper.

## Data Availability

The data that support the findings of this study are available from the corresponding author, upon reasonable request.

## Appendix Supplementary materials

Image, application 1
