# Supplementary material for: Physical performance, health-related quality of life and sleepiness severity of an adult outpatient population with narcolepsy: A cross-sectional analysis
Source: Int J Clin Health Psychol. 2025 May 2;25(2):100573. doi: 10.1016/j.ijchp.2025.100573 (PMC12124624; doi:10.1016/j.ijchp.2025.100573)
Supplement: Supplementary file 1 [file mmc1.docx]

**Supplemental Table 1: Threshold Values for Physical Performance According to Age and Gender**

| **Test** | **Age group** | **Male (n=10)** | | | **Age group** | **Female (n=12)** | | |
| --- | --- | --- | --- | --- | --- | --- | --- | --- |
|  |  | **Score** | **Percentile** | **Classification** |  | **Score** | **Percentile** | **Classification** |
| **Predicted VO_2_ Max, mL.kg.min-1** | 18-25 (n =7) | 38.2 | 30th | Below Average | 18-25 (n=5) | 48.2 | 35-40th | Below average |
|  | 26-35 (n=1) | 39.9 | 50-5th | Average | 26-35 (n=1) | 25.7 | 35-40th | Below average |
|  | 36-45 (n=1) | 30.9 | 35-40th | Below Average | 36-45 (n=4) | 33.4 | 40-45th | Average |
|  | 46-55 (NA) |  | | | 46-55 (n=1) | 34.3 | 65th | Above Average |
|  | 56-65 (n=1) | 39.6 | 55^th^ | Good | 56-65 (n=1) | 36.2 | 85th | Good |
|  | | | | | | | | |
| **Hand Dynamometry, kg** | **Age group** | **Score** | **Percentile** | **Norm** | **Age group** | **Score** | **Percentile** | **Norm** |
|  | 20-24 (n=5) | 42.2 | 50-75^th^ | 41.5 | 20-24 (n=4) | 22.0 | 10-25th | 28.4 |
|  | 24-29 (n=3) | 42.2 | 25-50^th^ | 48.8 | 24-29 (n=2) | 28.8 | 25-50th | 30.6 |
|  | 30-34 (NA) |  | | | 30-34 (n=1) | 25.2 | 10-25th | 31.4 |
|  | 35-39 (NA) |  |  |  | 35-39 (NA) |  | | |
|  | 40-44 (n=1) | 48.1 | 25-50th | 50.3 | 40-44 (n=2) | 28.6 | 25-50th | 30.7 |
|  | 45-49 (NA) |  | | | 45-49 (n=2) | 25.2 | 10-25th | 29.9 |
|  | 50-54 (NA) |  |  |  | 50-54 (NA) |  | | |
|  | 55-59 (n=1) | 36.9 | 10-25th | 46.2 | 55-59 (NA) |  |  |  |
|  | 60-64 (NA) |  | | | 60-64 (n=1) | 25.8 | 25-50th | 26.5 |
|  | | | | | | | | |
| **Peak Power/Body Mass, W/Kg** | **Age group** | **Score** | **Age-Matched Norm** | | **Age group** | **Score** | **Age-Matched Norm** | |
|  | 20-29 (n=8) | 46.0 | 59.2 | | 20-29 (n=6) | 38.5 | 43.3 | |
|  | 30-39 (NA) |  | | | 30-39 (n=1) | 36.6 | 41.9 | |
|  | 40-49 (n=1) | 31.3 | 46.5 | | 40-49 (n=4) | 40.7 | 39.9 | |
|  | 50-59 (n=1) | 37.7 | 44.9 | | 50-59 (NA) |  | | |
|  | 60-69 (NA) |  | | | 60-69 (n=1) | 40.8 | 27.9 | |
|  | | | | | | | | |
| **Press Up Repetitions, n** | **Age group** | **Score** | **Classification** | | **Age group** | **Score** | **Classification** | |
|  | 20-29 (n=8) | 17.8 | Fair | | 20-29 (n=6) | 12.7 | Fair | |
|  | 30-39 (NA) |  | | | 30-39 (n-1) | 6.5 | Needs improvement | |
|  | 40-49 (n=1) | 7.0 | Needs improvement | | 40-49 (n=4) | 8.3 | Fair | |
|  | 50-59 (n=1) | 4.0 | Needs improvement | | 50-59 (NA) |  | | |
|  | 60-69 (NA) |  | | | 60-69 (n=1) | 1.0 | Needs improvement | |
|  | | | | | | | | |
| **Wall Sit Duration, seconds** | **Age group** | **Score** | **Classification** | | **Age group** | **Score** | **Classification** | |
|  | 19-29 (n=8) | 70.4 | <25th Percentile | | 19-29 (n=6) | 70.9 | 25-50th | |
|  | 30-39 (NA) |  | | | 30-39 (n=1) | 32.5 | <25th Percentile | |
|  | 40-49 (=1) | 51.5 | <25th Percentile | | 40-49 (n=4) | 33.1 | 25-50th | |
|  | 50-59 (n=1) | 54.6 | 25-50th | | 50-59 (NA) |  | | |
|  | 60+ (NA) |  | | | 60+ (n=1) | 36.3 | 50-75th | |
